# The complete mitochondrial genome of the invasive cyanobacteriosponge *Terpios hoshinota* (Demospongiae, Suberitida, Suberitidae)

**DOI:** 10.1080/23802359.2023.2180311

**Published:** 2023-02-24

**Authors:** Zoe T. Richards, Hiroki Kise, Katrina M. West

**Affiliations:** a Coral Conservation and Research Group, Trace and Environmental DNA Laboratory, School of Molecular and Life Sciences, Curtin University, Bentley, Western Australia; b Collections and Research, Western Australian Museum, Welshpool, Western Australia; c Geological Survey of Japan, National Institute of Advanced Industrial Science and Technology, AIST Tsukuba Central 7, Tsukuba, Ibaraki, Japan; d Molecular Invertebrate Systematics and Ecology Laboratory, Graduate School of Engineering and Science, University of the Ryukyus, 1 Senbaru, Nishihara, Okinawa, Japan; e CSIRO Australian National Fish Collection, National Research Collections Australia, CSIRO, Hobart, Tasmania, Australia

**Keywords:** Invasive species, Illumina, mitogenome, phylogenetics, Porifera, Subclass Heteroscleromorpha

## Abstract

The cyanobacteriosponge *Terpios hoshinota* occurs on tropical reefs throughout the Indo-Pacific. The species encrusts live coral, and other benthos, and is considered a pest species that can threaten the health and productivity of locally native benthic communities on coral reefs. Here we assemble a complete mitochondrial genome to aid further research into the range expansion of this species. The circular genome was 20,504 bp in length and encoded 14 protein-coding genes, two ribosomal RNA (rRNA) genes, and 25 transfer RNA (tRNA) genes. A phylogenetic analysis based on the concatenated sequences of 14 protein-coding genes of 12 members of the subclass Heteroscleromorpha including the newly sequenced *T. hoshinota*, suggests further taxonomic revisions within the order Suberitida may be warranted.

## Introduction

*Terpios hoshinota* Rützler & Muzik, 1993 (Demospongiae, Suberitida, Suberitidae) is an encrusting marine sponge with a thin tissue layer (<1 mm) that can be grey, brown or black. This species is considered invasive and is commonly called the “black disease.” It is an aggressive space competitor capable of growing 1–2 mm per day (Liao et al. 2007). Its fast growth rate is likely due to the nutritional benefits of hosting a large quantity of non-photosymbiotic organisms. The sponge grows by lateral propagation, extending short fine tendrils across crevices to encounter new substrates. Thus, it advances as a sheet and can make bridges between branches of corals to aid its encroachment. It actively overgrows live or dead corals and other benthic fauna, including hydrozoans, octocorals, and Tridacnid clams leading to their suffocation and death. Since being discovered in Guam in 1971, new distribution records have documented the expansion of the species (Liao et al. 2007; Reimer et al. 2011; Fujii et al. 2011; Shi et al. 2012; de Voogd et al. 2013; Montano et al. 2014; Ekins et al. 2017; Fromont et al. 2019). This sponge is responsible for the demise of large reef areas, particularly in pollution-stressed nearshore zones (Rützler and Muzik, 1993), but it can also occur on relatively pristine reefs (Reimer et al. 2011; van der Ent et al. 2016; Fromont et al. 2019).

Whether the increased prevalence of *T. hoshinota* is a consequence of natural or artificial range expansion (possibly via shipping translocations) or easier recognition (and thus documentation) of the species is unclear. Existing genetic data indicates a moderate amount of haplotype diversity in the COI region (van der Ent et al. 2016; Fromont et al. 2019). Samples from the Kimberley (north-western Australia) shared a single haplotype with an Indonesian sample indicating the potential for a single point of introduction to Western Australia (Fromont et al. 2019). Further data with additional loci and population-level analyses are needed to confirm whether this species is undergoing rapid distribution expansion (Montano et al. 2014) and the source/directionality of introductions. The generation of a mitogenome for this species will aid such future studies.

## Materials

**Figure 1.**
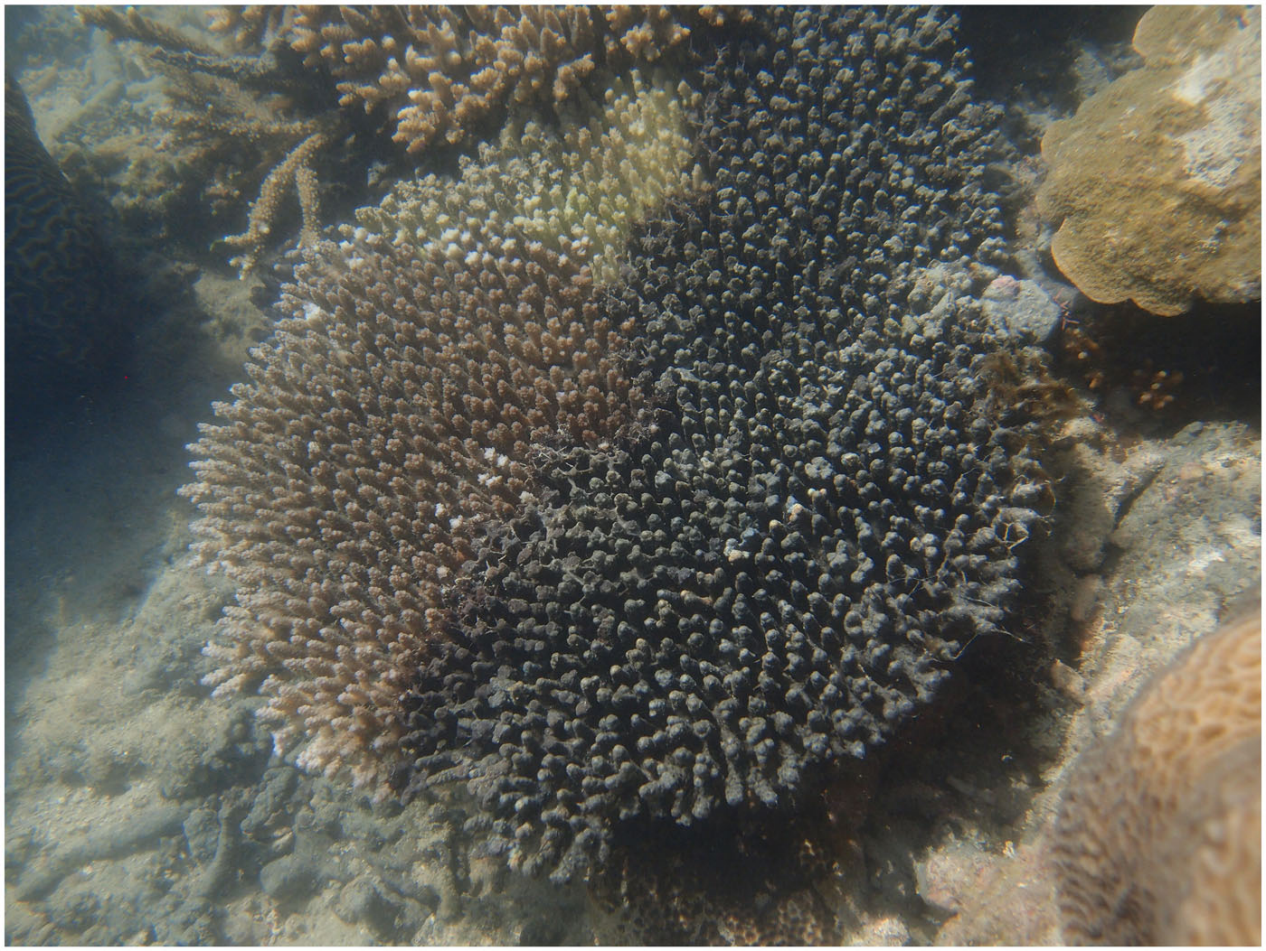
*Terpios hoshinota* overgrowing a corymbose *Acropora* at Berthier Island Kimberley, 2016. Photo by Zoe Richards.

A single specimen of *T. hoshinota* with accession number Z83368 from the Western Australian Museum Marine Invertebrate Collection was used in this study (https://museum.wa.gov.au/research/research-areas/aquatic-zoology, Contact Marine Invertebrate Curator Dr Zoe Richards, zoe.richards@museum.wa.gov.au). The specimen was collected from Berthier Island Kimberley (S14.50409 E124.98207) on the 21 September 2016 (See Figure 1).

## Methods

Total genomic DNA (gDNA) was extracted from tissue using the QIAGEN blood and tissue kit (Qiagen; Venlo, Netherlands), following the manufacturer’s protocol. The overall yield and quality of gDNA were measured using a Qubit fluorometer (Thermo Fisher Scientific, Waltham, MA, USA) and by electrophoresis on a 2% agarose gel stained with GelRed (Fisher Biotec, Wembley, WA, Australia). A Nextera Flex DNA library kit (Illumina Inc., San Diego, CA, USA) was used to assemble the gDNA library with a target gDNA input of 100 ng to saturate the tagmentation beads. The ligation efficiency was assessed via quantitative PCR (qPCR) using the JetSeq Hi-ROX kit (Bioline; Australia), containing an SYBR-based qPCR mix that targets Illumina’s P5 and P7 adaptors for sequencing. DNA standards ranging from 10 pM to 100 aM provide a quantitative value of the successfully ligated product. The library build was pooled in equimolar ratio, based on the JetSeq qPCR quantification results, with other library builds to produce a final genome library of 24 samples. The final genome library was size-selected for 200–600 bp size fragments using a Pippin Prep (Sage Sciences; USA), purified with the QIAquick PCR purification kit (Qiagen) and quantified with a Qubit fluorometer (Invitrogen; USA) in preparation for sequencing. The genomic library (2.1 pM load concentration) was sequenced on an Illumina NextSeq (Illumina, San Diego, CA, USA) with a High Output 300-cycle V2.5 chemistries (151 bp paired-end sequencing) following the manufacturer’s protocol.

The mitogenome was assembled using NOVOPlasty (Dierckxsens et al. 2017), with an input seed of a COI region of *Terpios hoshinota* (GenBank accession number MN507878.1). The mitogenome was primarily annotated using MITOS (Bernt et al. 2013; genetic code: 4) and the reference sequence MN507878.1 in Geneious v10.0.6 (Kearse et al. 2012). Transfer RNA genes were identified using the tRNAscan-SE v2.0 (Chan et al. 2021) and MITOS.

The online server Proksee (https://proksee.ca) that used GCview (Stothard and Wishart 2005) was used to generate the circular mitochondria genome map. The phylogenetic tree was reconstructed using Maximum-likelihood (ML) with 1000 bootstrap replicates in RAxML-NG (Kozlov et al. 2019) based on nucleotide sequences of 14 protein-coding genes from the mitogenomes of 11 other heteroscleromorphan species (Genbank accession numbers listed in Figure 3). All nucleotide sequences were aligned with MAFFT (Katoh and Standley 2013). ModelTest-NG v0.1.6 (Darriba et al. 2020) was used to select the best-fitting model for each protein-coding gene.

## Results

**Figure 2.**
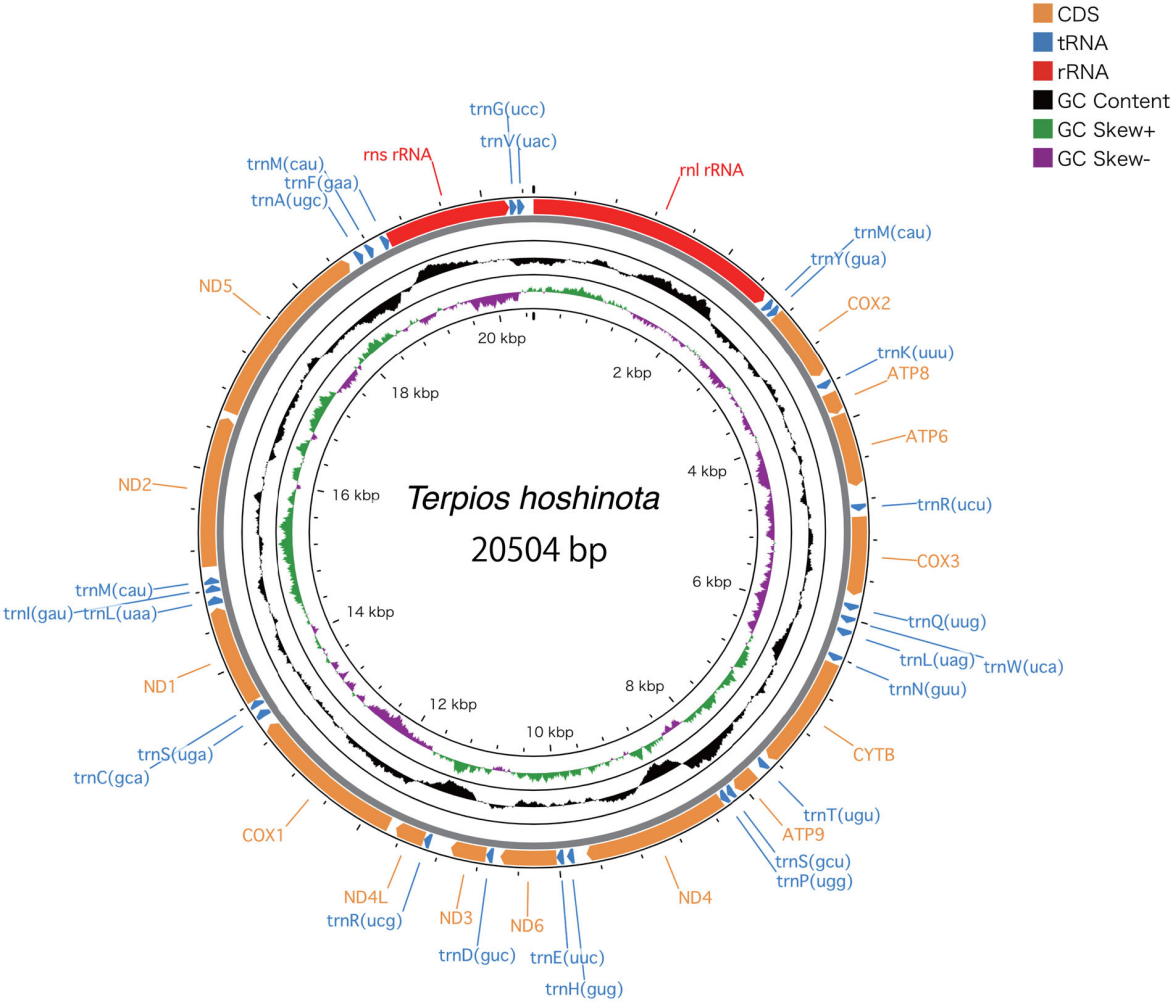
Mitochondrial genome architecture of *Terpios hoshinota*. Position of protein coding sequence (CDS) genes, rRNAs, and tRNAs are shown. GC content is plotted with a black sliding window, and GC skew is indicated by colored sliding window (green and purple color).

NOVOPlasty recovered a circularized contig of 20,504 bp in length, with an average coverage of 110x (Figure 2). The resulting annotation of the complete mitogenome consists of 14 protein-coding genes, 25 tRNA genes and two rRNA genes. Trn anticodons were further annotated to include their one-letter IUPAC amino acid abbreviation and to help distinguish isoacceptor genes. trnM is repeated three times, trnR, trmL and trnS are duplicated. All trnM genes were annotated as functional genes rather than pseudogenes as no pseudogenes were identified by tRNAscan-SE. The mitochondrial base composition was A 29.1%, C 14.8%, G 21.7%, T: 34.4%. The newly reported mitogenome was deposited in GenBank under accession number ON099442.1.

**Figure 3.**
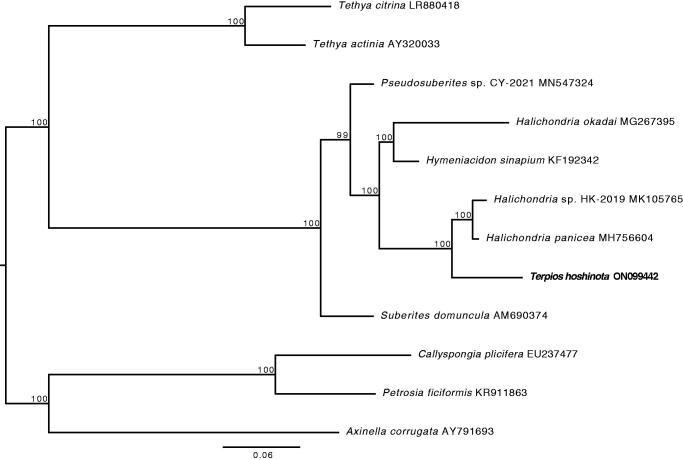
Phylogenetic analysis of the subclass Heteroscleromorpha based on the concatenated sequences of 14 protein-coding genes. The newly sequenced *T. hoshinota* mitogenome is highlighted in bold. Numbers above the branches indicate ML bootstrap values from 1,000 replicates.

The phylogenetic reconstruction of the subclass Heteroscleromorpha based on the concatenated sequences of 14 protein-coding genes was strongly supported at all nodes including the node connecting *T. hoshinota* to *Halichondria* (Figure 3).

## Discussion and conclusion

The mitochondrial genome of the Suberitid sponge *Terpios hoshinota* was 817–1027 base pairs longer than those published for the *Halichondria* sister group (Knobloch et al. 2019; Kim et al. 2019). The length of these Heteroscleromorphan mitogenomes is 2,000–3,000 base pairs longer than those of Hexasterophoran sponges (see *Aphrocallistes vastus*, Rosengarten et al. 2008). The mitogenome architecture followed the typical Demospongidae arrangement (Salas-Castañeda et al. 2019), and the nucleotide composition had high content of A + T (63.5%) similar to other Suberitid sponges (Kim et al. 2019).

The protein-coding genes were typical of Demospongidae including the presence of *apt9* (Zardoya 2020). A synteny putatively ancestral to the metazoans, *cox2-apt8-apt6-cox3*, is retained. A gene sequence of *cox1-tRNA(s)-nad1* which is shared among the demosponges is also present; however, *tRNA(c)* also appears in this gene block in *T. hoshinota.* The complement of 25 tRNA genes is larger than that recorded in some demosponges and hexactinellids (20–22, Haen et al. 2007) but identical to that of *Halichondria* (Kim et al. 2017, 2019). The difference is accounted for by repeats of trnM, trnR and trnS in *T. hoshinota*.

The phylogenetic reconstruction showed *T. hoshinota* (family Suberitidae) groups with halichondrids instead of the suberitids (represented by *Suberites domuncula* in Figure 3) indicating polyphyletic relationships between the families Subertitidae and Halichondriidae. The absence of monophyly within these two families has previously been observed through 28S nuclear-ribosomal genes (see Thacker et al. 2013) and is consistent with the current understanding of relationships within the Subclass Heteroscleromorpha (Morrow and Cardenas 2015). This mitogenomic dataset adds to the body of evidence suggesting further taxonomic revisions within the order Suberitida may be warranted. However, further molecular systematic analyses utilizing broader next-generation datasets are needed to test if the relationships between mitogenomes reflect evolutionary relationships between species, and to underpin robust phylogenetic inferences about *T. hoshinota* and its relatives.

When occurring in outbreak proportions, *T. hoshinota* poses a threat to coral reefs across the Indo-West Pacific. The mechanisms responsible for *T. hoshinota* outbreaks remain uncertain; however, the propensity for long-range dispersal has helped this species expand its range (Chow et al. 2022). This new mitogenome provides a valuable genetic resource to help examine phylogenetic relationships within the Heteroscleromorpha. It also provides a foundation for expanding knowledge of *T. hoshinota* diversity and migration patterns across the Indo-West Pacific and may prove beneficial to unraveling what triggers outbreaks of this species.

## Data Availability

The genome sequence data that support the findings of this study are openly available in GenBank of NCBI at https://www.ncbi.nlm.nih.gov/ under accession no. ON099442.1. The associated BioProject, SRA, and BioSample numbers are PRJNA826711, SRR18788998, and SAMN27578314, respectively.
